# The Effect of High Lignin Content on Oxidative Nanofibrillation of Wood Cell Wall

**DOI:** 10.3390/nano11051179

**Published:** 2021-04-29

**Authors:** Simon Jonasson, Anne Bünder, Linn Berglund, Magnus Hertzberg, Totte Niittylä, Kristiina Oksman

**Affiliations:** 1 Division of Materials Science, Department of Engineering Sciences and Mathematics, Luleå University of Technology, SE 97187 Luleå, Sweden; simon.jonasson@ltu.se (S.J.); linn.berglund@ltu.se (L.B.); 2 Umeå Plant Science Centre, Department of Forest Genetics and Plant Physiology, Swedish University of Agricultural Sciences, SE 90187 Umeå, Sweden; anne.bunder@slu.se (A.B.); totte.niittyla@slu.se (T.N.); 3 SweTree Technologies AB, SE 90403 Umeå, Sweden; magnus.hertzberg@swetree.com; 4 Department of Mechanical & Industrial Engineering, University of Toronto, Toronto, ON M5S 3G8, Canada

**Keywords:** cellulose nanofibrils, wood, lignin, TEMPO-oxidation

## Abstract

Wood from field-grown poplars with different genotypes and varying lignin content (17.4 wt % to 30.0 wt %) were subjected to one-pot 2,2,6,6-Tetramethylpiperidin-1-yl)oxyl catalyzed oxidation and high-pressure homogenization in order to investigate nanofibrillation following simultaneous delignification and cellulose oxidation. When comparing low and high lignin wood it was found that the high lignin wood was more easily fibrillated as indicated by a higher nanofibril yield (68% and 45%) and suspension viscosity (27 and 15 mPa·s). The nanofibrils were monodisperse with diameter ranging between 1.2 and 2.0 nm as measured using atomic force microscopy. Slightly less cellulose oxidation (0.44 and 0.68 mmol·g^−1^) together with a reduced process yield (36% and 44%) was also found which showed that the removal of a larger amount of lignin increased the efficiency of the homogenization step despite slightly reduced oxidation of the nanofibril surfaces. The surface area of oxidized high lignin wood was also higher than low lignin wood (114 m^2^·g^−1^ and 76 m^2^·g^−1^) which implicates porosity as a factor that can influence cellulose nanofibril isolation from wood in a beneficial manner.

## 1. Introduction

The valorization of wood and other lignocelluloses generally include a variety of costly mechanical-chemical refining steps in order to overcome the inherent recalcitrance that comes from lignified cell walls and complex inaccessible crystalline cellulose microfibril architectures [1]. Effectively nanofibrillating the cell wall is an attractive prospect where cellulose nanofibrils (CNFs) are attainable as a final product or as an intermediate for further treatments that utilizes the increased accessibility [2]. For instance, through improved saccharification of nanofibrils compared to initial wood fibers [3]. An important factor that has made CNF isolation more favorable is the implementation of various pretreatments in addition to traditional pulping processes. These swell the cell wall structure and reduces its structural integrity, thereby making it more susceptible to complete nano-fibrillation. The choice of pretreatment, cellulosic feedstock and mechanical disintegration method varies across literature and is a subject of intense research activity [4]. A common process in this context is that of 2,2,6,6-Tetramethylpiperidin-1-yl)oxyl (TEMPO) catalyzed oxidation. This pretreatment mediates nanofibrillation through selective carboxylation of the cellulose C_6_-hydroxyl groups through N-oxyl radical generation [5]. The treatment has been studied in great depth in relation to delignified pulps from a variety of sources [6]. It has also been demonstrated that feedstock containing lignin, such as thermomechanical pulp [7], hemp [8] and wood [9], can be simultaneously delignified and carboxylated using the treatment. The relation between these procedures and nanofibrillation is complex where CNFs with residual lignin are attainable despite substantial carboxylation [10,11]. It has also been reported that the presence of the TEMPO-catalyst significantly increases carboxylation whilst keeping the delignifying efficiency constant [7]. The understanding of how these phenomena occurs in relation to native wood structures with varying lignin is currently unexplored. Available literature revolves around already processed feedstocks or focuses on pure mechanical fibrillation [12]. A natural step would thus be to combine TEMPO-oxidation with variation in lignin content of unprocessed feedstocks. The associated novelty includes highlighting TEMPO-oxidation as a metric of fibrillation suitability for raw wood with variation in lignin content, something which could be applicable to commercial endeavors where TEMPO-oxidation is an attractive prospect in obtaining CNFs. Direct TEMPO-oxidation of wood has only recently been the subject of few dedicated studies [9,13]. These studies were furthermore showcasing one type of wood whereas the study presented herein aims to explore an additional factor of wood characteristics. This can increase the knowledge and feasibility of direct oxidation of wood by elucidating how wood properties, in this case native lignin content, influences the process.

In this study, we investigated field-grown poplar trees with large (17.4–30.0 wt %) systematic differences in lignin content. The goal was to analyze how lignin content associated wood recalcitrance is manifested in relation to nanofibrillation after pretreatments that simultaneously delignifies and carboxylates the wood fibers. Increased understanding of how oxidative treatments interplay with variation in lignin content of the feedstock is beneficial to a wide range of CNF isolation endeavors through elucidation of the link between wood and final CNFs.

## 2. Materials and Methods

### 2.1. Materials

Six different wood samples with specific chemical composition (see Table 1) were supplied by SweTree Technologies AB, Umeå Sweden. The samples were selected out of a library of trees (see Supplementary Material) based on the largest variety of total lignin content as determined using pyrolysis GS/MS [14]. The variation was validated using principal component analysis (see Supplementary Material). Three wood samples with similar lignin content were chosen as center points, approximately corresponding to lignin content in between the lowest and the highest samples. This was done in order to be able to evaluate trends between lignin content and characterization if present.

High-purity sodium chlorite (77.5–82.5%), standard hydrochloric acid solution (0.5 N), standard sodium hydroxide solution (0.1 N), sodium hydroxide beads (≥97%, ACS) were purchased from VWR, Solna, Sweden. TEMPO (99%), sodium hypochlorite (NaClO, 6–14% active chlorine) and Congo red (analytical standard) were purchased from Sigma-Aldrich, Stockholm, Sweden AB. All chemicals were used as received.

### 2.2. Oxidation and Homogenisation

Wood powder of the grinded poplar trees were sieved through a 300–500 µm mesh prior to sampling (2 g) and soaking in (100 mL) distilled water for 24 h prior to further treatment. Primary oxidant sodium chlorite 5.0 g·g^−1^ wood and TEMPO (17.5 mg·g^−1^ wood) was added together with a phosphate buffer (0.1 M, 100 mL, pH = 6.8) to the vessel (250 mL) containing the wet powder. Each vessel with respective suspension was closed and submerged into a SBS40 shaking water bath (Cole-Parmer Stuart, Staffordshire, United Kingdom) at 60 °C for 1 h at 200 rpm in order to dissolve NaClO_2_ and TEMPO. Shaking water bath was used to eliminate experimental error from variation in heating rate and stirring and to make sure each tree sample was subjected to identical processing conditions. The reaction was initiated through addition of sodium hypochlorite (2 mL·g^−1^ wood) and then left for 48 h. The methodology was adapted from [16] with changes to the amount of primary oxidant and feedstock characteristics. The fully delignified and carboxylated wood was filtered thoroughly and then homogenized one pass without recirculation using an APV-2000 high-pressure homogenizer (SPX Flow Inc, Silkesborg, Denmark) with an average flow rate of 4 mL·s^−1^ at a pressure of 1000 bar. The concentration of respective sample was adjusted prior to and after homogenization to eliminate unwanted effects from variation in solid content.

### 2.3. Viscosity of CNF Suspensions

The viscosity of the oxidized and homogenized suspensions was measured at the same concentrations (0.183 ± 0.01 wt %) using a Vibro Viscometer (SV-10, A&D Company Limited, Tokyo, Japan) with a tuning fork vibration method at a vibrational frequency of 30 Hz. The measurements were repeated three times at room temperature for each sample.

### 2.4. Carboxylate Content

Carboxylate content was analyzed through the electric conductivity titration method adapted from [17]. 150 mL of respective CNF (cellulose nanofibril) suspensions (≈0.2 wt %) were protonated through addition of 0.1 M hydrochloric acid and 0.01 M sodium chloride. The suspensions were titrated with fresh 0.01 M sodium hydroxide through 0.5 mL increments until pH 10 was reached. The amount of carboxylate groups (mmol·g^−1^) induced from TEMPO-oxidation was calculated through Equation (1) below where c is the concentration of the sodium hydroxide, V_2_ and V_1_ is the volume of added sodium hydroxide at the end and start, respectively, m is the mass of the cellulosic material in the sample, calculated through subtraction of added acid, base and salt mass from the oven dried suspension. The titration was repeated three times.

(1) $$\mathrm{Carboxylate}\;\mathrm{groups}\;\left(\frac{\mathrm{mmol}}{g}\right)=\frac{C\left(V_{2}-V_{1}\right)}{m}$$

### 2.5. Atomic Force Microscopy

Tapping-mode atomic force microscopy (AFM) Veeco MultiMode scanning probe, Santa Barbara, CA, USA, was used to confirm the presence of nanofibrils and analyze the morphology. Antimony-doped silicon cantilevers (TESPA-V2, Bruker, Camarillo, CA, USA) with a spring constant of 42 N·m^−1^ and a nominal tip radius of 8 nm were used for the analysis. Samples were prepared by depositing a small droplet of the CNF suspension (0.001 wt %) on a freshly cleaved mica plate and letting it air dry for ≥ 5 h. Around 100 fully individualized nanofibrils, total from four micrographs were analyzed for each sample and presented as a mean. 

### 2.6. Process and Nanofibril Yield

Process yield was calculated as the washed gravimetric yield after the chemical treatment relative dry wood mass. The fraction of the process yield that comprised individual, colloidally stable nanofibrils were further quantified through centrifugation of the suspensions at 12,000× *g* (Avanti J25i, Beckman Coulter Inc. Brea, CA, USA) for 20 min at an approximate consistency of 0.2 wt %. The suspensions were decanted and the solids retained in the sediment were dried for 24 h at 95 °C. This was repeated three times and the nanofibril-fraction was then calculated according to the Equation (2), where m_p_ and m_s_ is the dry precipitate and supernatant mass in the sample, respectively. The nanofibril-fraction (Φ) was presented as fraction of the process yield.

(2) $$\Phi =1-\frac{m_{p}}{m_{p}+m_{s}}$$

### 2.7. Thermogravimetric Analysis

Dried CNF suspensions were subjected to thermogravimetric analysis TGA-Q500, TA Instruments, (New Castle, DE, USA) to analyze the relative amount of residue after pyrolysis of the oxidized wood samples and to compare the thermal stability of the CNFs. A heating rate of 10 °C·min^−1^ from room temperature to 900 °C was used in a nitrogen atmosphere.

### 2.8. Klason Lignin and Cellulose Content

Cellulose content of the wood holocellulose and dried CNF suspensions were calculated based on extraction using 17.5 M NaOH according to TAPPI-standard [15], where the oven dried gravimetric yield after filtration was used to estimate α-cellulose content. Klason lignin for the dry CNF suspensions was analyzed according to standard [18] through hydrolysis using sulfuric acid

### 2.9. Network Manufacturing

CNF suspensions were degassed for 30 min in a vacuum oven prior to vacuum filtration on hardened filter paper (Whatman, Grade 52, GE Healthcare, Machelen, Belgium, Pore size: 7 µm). The wet networks were carefully peeled from the filter paper and dried to around 14 % solid content prior. The stable networks were placed between two blotting papers and pressed (2 kN·m^−2^) for 10 h. The dry networks were further compression molded using Fontijne Grotnes LPC-300 (Vlaardingen, Netherlands), between two mylar films (Lohmann Technologies, Knowl Hill, UK) at a pressure of 0.32 MPa and at a temperature of 120 °C. 

### 2.10. X-ray Diffraction

An X-ray diffractometer (PANalytical, Almelo, Netherlands) equipped with a PIXcel^3d^ detector was used to measure the degree of crystallinity of the dried nanofibrils. Cu-Kα radiation (λ = 0.154 nm) was used during analysis with operation voltage of 45 kV and a current of 40 mA. The crystallinity index (CI) was calculated according to the peak height method after baseline correction [19]. This was done according to Equation (3), where I_200_ is the intensity of the crystalline (200) peak at 2θ = 22.4 and I_am_ is the intensity minimum between nearby crystalline peaks at 2θ = 18.

(3) $$\mathrm{CI}\;\left(\%\right)=\frac{I_{200}-I_{\mathrm{am}}}{I_{200}}\times 100$$

### 2.11. Mechanical Testing

The dry networks were cut into rectangular samples (40 mm × 5 mm) using a mechanical punch. The samples were then stored at 50% RH for at least two days prior to mechanical characterization. Mechanical testing was performed using a Shimadzu AG-X universal testing machine (Kyoto, Japan) with a 500 N load cell. Testing was performed at a crosshead speed of 10%·min^−1^, with the strain being measured using a video extensometer (high-speed camera, HPV-X2). The gauge length was set to 20 mm for each measurement. Seven specimens were analyzed for each network batch. The tensile strength was reported as the maximum strength at break. Young’s modulus was calculated from the slope of the stress–strain curve at early strain (0.1–0.5 %). 

### 2.12. Sample Porosity and Moisture Content

Density of the networks were measured by calculation based on the specimen volume and weight. Porosity (P) was derived using Equation (4), where ρ_s_ and ρ_c_ is the density of the specimen and cellulose (1.5 g·cm^−3^), respectively [20] Moisture content of the specimen was calculated from difference in weight after 24 h in oven at 105 °C

(4) $$P=1-\frac{\rho_{s}}{\rho_{c}}$$

### 2.13. Wood Porosity Analysis

Intact wood samples at the extreme values of initial lignin content (17.4 and 30.0%) were oxidized in an identical manner as described in Section 2.2. The oxidized samples were carefully washed by submerging the samples into 20 L of distilled water. The fragile delignified and carboxylated samples were left to soak for four hours and the process was repeated three times with fresh distilled water. Brunauer–Emmett–Teller (BET) specific surface area analysis of the samples were performed by first freezing the wet samples at −20 °C for 10 h prior to freeze-drying for 72 h using Alpha 1-2 LD plus (Martin Christ Gefriertrocknungsanlagen GmbH, Osterode am Harz, Germany). The samples were degassed overnight at 120 °C followed by N_2_ physiosorption using a Micromeritics Gemini VII surface area analyzer (Norcross, GA, USA). A total of 30–60 mg of oxidized wood sample was analyzed for a respective sample.

Surface area of the oxidized wood was also estimated in the wet state by analyzing congo red absorption of the cellulosic fibers as described by [12]. Briefly, the absorbed dye was quantified using a UV-vis spectrophotometer (GENESYS, 10 UV, Thermo Scientific, Schwerte, Germany) at the absorption maxima 500 nm and then translated to surface area (SSA) using Equations (5) and (6), where E is the solution concentration of congo red at equilibrium (mg·mL^−1^), A_max_ is the absorbed amount of congo red on the sample (mg·g^−1^), K_ad_ is the equilibrium constant, N is Avogadro’s constant, SA is the theoretical surface area of a congo red molecule (1.73 nm^2^) and MW is the molecular weight of congo red (697 g·mole^−1^).

(5) $$\frac{E}{A}=\frac{1}{K_{\mathrm{ad}}A_{\max}}+\frac{E}{A_{\max}}$$

(6) $$\mathrm{SSA}=\frac{A_{\max}\times N\times \mathrm{SA}}{\mathrm{MW}\times 10^{21}}$$

Samples cut in the wet state were freeze-dried and analyzed using a scanning electron microscope JEOL (JSM-IT300, Tokyo, Japan) at an acceleration voltage of 5 kV. A 15 nm layer of platinum was sputtered on the samples prior to analysis.

## 3. Results and Discussion

### 3.1. Fibrillation Efficiency and Nanofibril Characteristics

**Figure 1:**
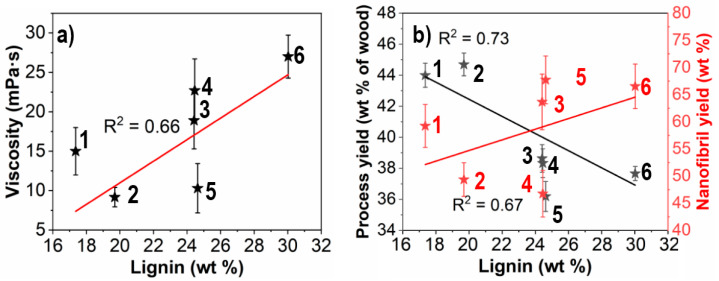
Viscosity (**a**), process yield (**b**) and nanofibril yield (b, red) of CNF suspensions obtained from wood with varying initial lignin content. Data represent mean ± SD of six different CNF samples (*n* = 3 replicates per sample). Data points for respective samples indicated with black and red stars.

Suspensions of oxidized and homogenized wood samples showed a difference in viscosity between 9 and 27 mPa·s (shown in Figure 1a) is indicative of a qualitatively higher degree of fibrillation [21]. A similar behavior is reflected in the fraction of the yield that comprises fine colloidally stable nanofibrils, (shown in Figure 1b). A total of 45–68 wt % of the yield was recovered upon fractionating. A higher recovery of nanofibrils from wood with higher lignin content can be seen which is supported by the increased viscosity. The process yield ranged between 36 and 44 wt % of dry initial wood and decreased with more initial lignin content of the wood. 

This can be understood from the complete dissolution of the lignin after being subjected to the catalytic conditions in this study. Klason lignin difference was also negligible after the treatment where all samples had an estimated residual lignin content of <1 wt %, indicating that a full dissolution of lignin took place following oxidation. A similar enhanced delignification has been observed after treatment of thermomechanical pulp using the related TEMPO/NaBr/NaClO-system [7]. It is further noteworthy that the nanofibril yield relative initial dry wood is similar for high lignin containing wood despite lower relative cellulose content. This is indicative of effects associated with higher initial lignin content and is the subject of discussion in Section 3.3.

**Figure 2:**
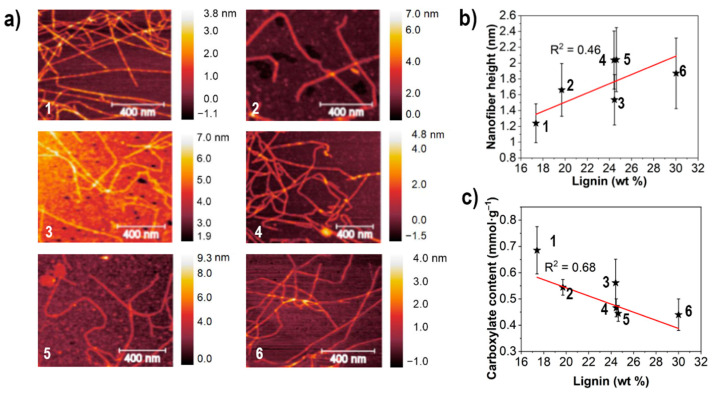
AFM micrographs (**a**) of the CNFs with corresponding height (**b**) and carboxylate content (**c**). Error bars in (**b**) derived from standard deviation of the size distribution. Data represent mean ± SD of six different CNF samples with 50–100 measurements per sample. Data points for respective samples indicated with black stars.

Carboxylate content of the final suspensions is shown in Figure 2 together with AFM micrographs of the CNFs that comprises the suspensions. Carboxylate content ranged between 0.44 and 0.68 mmol·g^−1^ of cellulose and was higher for low lignin wood compared to high. The range of the height of the individualized CNFs was measured between 1.2 and 2.0 nm and are thus thinner than most models suggest for elementary fibrils of wood cellulose. Significant differences in height were only apparent when comparing low lignin wood (sample #1) to the rest.

The relation between carboxylate content and initial lignin content indicates a decrease in carboxylation with more initial lignin of the feedstock although the correlation cannot be extrapolated to a larger range of carboxylation due to a rather narrow interval that the carboxylation ranges within. Interestingly, at 0.37 mmol·g^−1^ it has been calculated that all the surfaces of native cellulose microfibrils have undergone oxidation (Sjöstedt, 2014), which implies that the smaller individual elementary fibrils comprising these microfibrils are the subjects of a variable oxidation throughout this study. This can be explained by looking at the oxidation process where lignin hydroxyl groups are efficiently oxidized and depolymerized by TEMPO-systems through cleaving of C-C and ether bonds [22]. Lignin is present in both the thin middle lamella and the cell wall layers of wood [23] and can expectedly be preferentially oxidized during the process. This would decrease the effectiveness of the cellulose oxidation since lignin is more accessible than cellulose and the process is defined by a specified amount of chlorite (5 g·g^−1^ wood). This has been reported previously [24] and explained through splitting of the elementary fibril into smaller nanofibrils and even individual polymer chains [25]. This fragmentation phenomenon has been reported by multiple authors [24,25,26,27]. The increased carboxylation taking place for the cellulose derived from low lignin wood would according to this hypothesis be related to the decreased height of the CNFs from low lignin wood as shown in Figure 2. The correlation between the size distribution as obtained from AFM and other metrics of fibrillation is furthermore more accurate for highly fibrillated samples as in this study [28].

**Figure 3:**
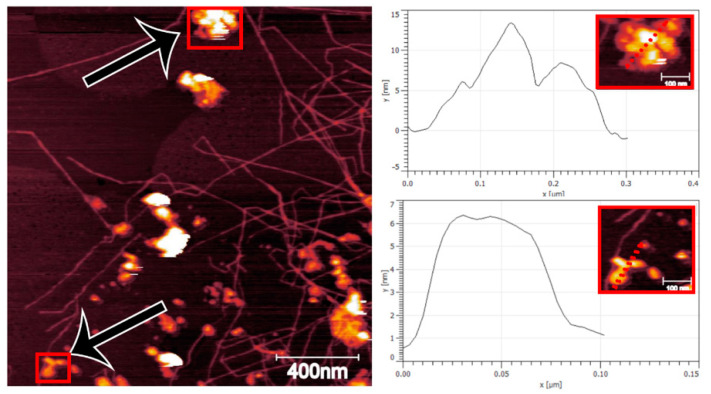
AFM micrograph of CNF (**left**) made from high lignin wood with particles and clusters of irregular shapes highlighted (**right**).

From the AFM analysis there were also particles present across the samples, especially apparent in CNFs made from high lignin wood. A larger scale AFM micrograph of that sample is shown in Figure 3, where particles and clusters are scattered among the CNFs. 

**Figure 4:**
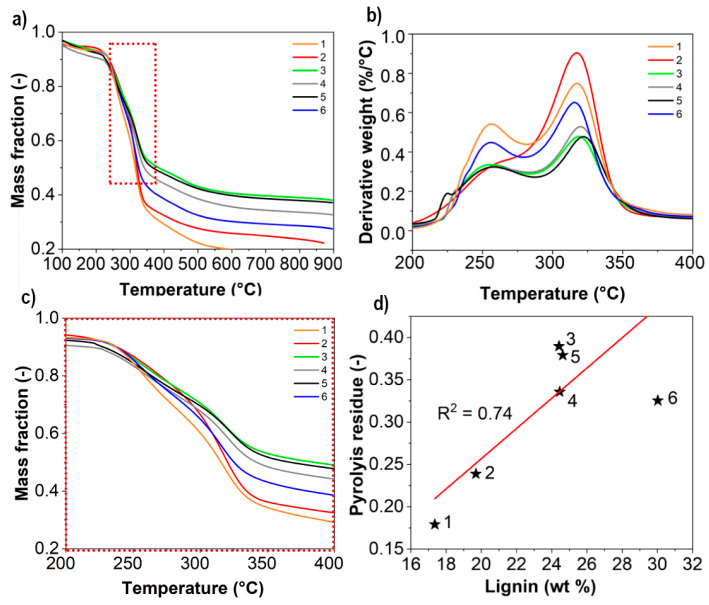
Thermal degradation curves showing mass loss (**a**), derivative weight (**b**) mass loss curve with zoom-in (200–400 °C) (**c**) and corresponding mass fraction of residues remaining at 900 °C (**d**) Data points for respective samples indicated with black stars.

This is hypothesized to be residues from processing. Even though extensive filtration took place during the isolation process it is likely that trapping of non-cellulosic components occurs inside the cell wall during oxidation and liberated together with the CNFs during homogenization. This is further supported by an increase in char after pyrolysis of the samples as shown in Figure 4. A mass fraction between 0.16 and 0.38 was remaining after pyrolysis of nanofibrils with more residues remaining for CNFs made from high lignin wood. The thermal stability of all CNFs was also similar as shown in Figure 4 and this is in agreement with what commonly is reported for CNFs derived from the TEMPO/NaClO/NaClO_2_-treatment.

Pyrolysis of lignin generally leads to a higher char formation compared to cellulose and hemicellulose [29]. In the absence of lignin, however, as shown for these samples, the residues have to originate from another source. Higher amount of char formation from the pyrolysis of deprotonated CNFs have been reported, where a difference up to 16 wt % was reported solely from the deprotonation of the carboxylate groups [30]. This effect together with the presence of residues from the oxidation explains this phenomenon and is further elaborated upon in Section 3.3 with porosity analysis of the TEMPO-oxidized wood. Thermal degradation occurs earlier compared to chlorite bleached wood, but later compared to cellulose that has undergone the harsher TEMPO/NaBr/NaClO-treatment [27]. The lack of variation amongst the samples is also supported by the carboxylate content (Figure 2), where the differences are relatively small in comparison to the whole range of carboxylate content that is possible to obtain after TEMPO-oxidation of all available hydroxyl groups on the nanofibril surfaces.

### 3.2. Dry Network Characteristics

**Figure 5:**
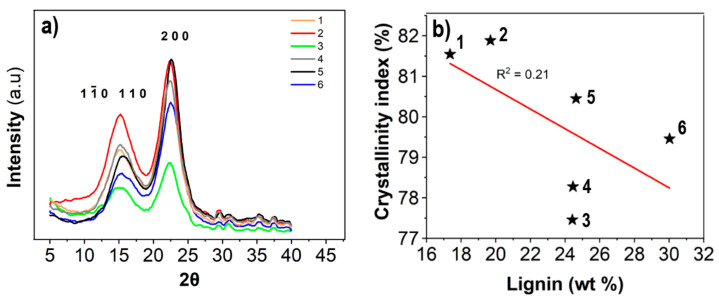
X-ray diffractograms of dry CNF networks (**a**) with corresponding Segal crystallinity indices (**b**). Data points for respective sample are indicated with black stars.

X-ray diffractograms of dried networks and corresponding degree of crystallinity is presented in Figure 5. The degree of crystallinity of the networks was similar at a range between 77 and 82% with a slight decrease from low to high lignin wood. The native cellulose I_β_ crystal structure was retained after processing for all samples, as indicated by the diffractogram peak location [31]. Both the absolute value of crystallinity and tendencies for a decrease in crystallinity as a function of fibrillation agree with previous analysis of a wide range of CNFs [32].

The presence of moieties responsible for variation in char formation (as shown in Figure 4) seems to have no effect on crystallinity. This is in accordance with the general behavior of TEMPO-oxidized celluloses [17] and is supported by reports that indicate that degree of crystallinity is not related to the relative amount of cellulose in a given sample [30].

**Figure 6:**
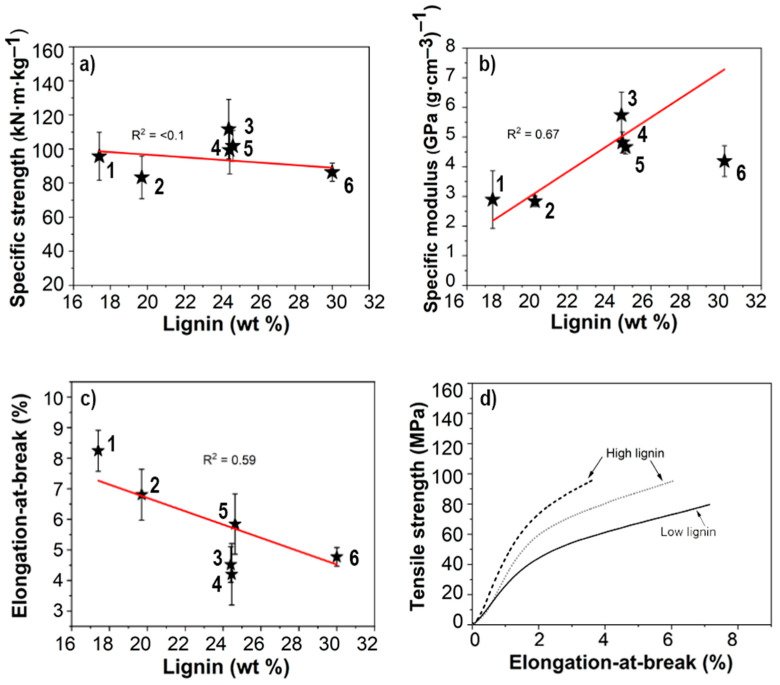
Mechanical properties of dry CNF networks with specific strength (**a**), specific modulus (**b**) and elongation-at-break (**c**). Representative stress–strain curves that showcase the difference between networks made from low and high lignin wood (**d**). Data represents mean ± SD of six different network samples (*n* = 7 replicates per sample). Data points for respective sample are indicated with black stars.

The mechanical properties of the networks are presented in Figure 6 and shows specific strength that is constant with respect to lignin content. A slight increase can be seen for the middle samples, but not indicative of any trend. Specific modulus increased with initial lignin content between 3 and 6 GPa, whereas the elongation-at-break decreased from 8.2% to 4.2%. The opposite trends for modulus and elongation-at-break are noteworthy and is indicative of the presence of previously mentioned impurities. The presence of stiff particles would significantly increase the stiffness of the networks whilst at the same time decreasing the elongation-at-break by acting as defects and limiting stress transfer between the CNFs [33].

Additional characteristics that are measurable and may influence the behavior of the dry networks are porosity/density, moisture content and relative amount of cellulose [20] This data is presented in Table 2 and shows that there are no obvious differences in physical characteristics of the specimen that may have been responsible for skewing the mechanical properties of the dry networks. The presence of a relatively large fraction of hemicellulose (24–31 wt %) is also interesting and is in agreement with our previous reports on similar processing for poplar wood [27] where an α-cellulose content of 70 wt % was obtained after direct TEMPO/NaClO/NaClO_2_ treatment. 

### 3.3. Wood Porosity Hypothesis

The results shown in this work have been argued in part on the basis of a higher porosity in the wood cell wall after oxidation of samples with a larger amount of initial lignin. Entrapment of subsequent treatment chemicals within the porous cell wall have also been connected to the increased amount of char following pyrolysis despite negligible amount of residual lignin. In order to substantiate this hypothesis further, intact pieces of wood were subjected to similar oxidation processes and investigated in terms of surface area following TEMPO-oxidation and electron microscopy of the wood surfaces. The surface area as estimated from BET and congo red adsorption is presented in Table 3 and indicates a larger surface area in the wet state (congo red) for high lignin wood (114 and 76 m^2^g^−1^) compared to low. The difference between both samples is negligible after freeze-drying and is lower compared to the wet state (4.4 and 6.9 m^2^g^−1^). 

The large difference in surface area estimated from BET and congo red adsorption can be explained by significant hornification occurring during freeze-drying and subsequent blocking of pores across different length scales whereas dye adsorption is performed for never-dried samples. Similar, relatively low values (14–42 m^2^g^−1^) have been reported for BET analysis of CNF aerogels [34], which was explained through irreversible agglomeration of the cellulosic surfaces during removal of water (hornification). A higher estimated surface area for dye adsorption compared to BET has also been reported for CNCs [35]. Hornfication for CNFs is, furthermore, more pronounced compared to CNCs [36], which further supports the increased differences observed in this study.

**Figure 7:**
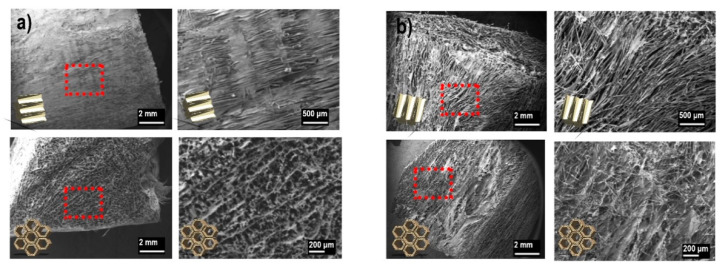
Structure of TEMPO-oxidized wood samples with low lignin wood (**a**) and high lignin wood (**b**). Upper figures showing the structure in the direction of the wood fibers and the lower showing corresponding cross sections.

SEM micrographs of oxidized wood sections are shown in Figure 7 and further supports the observation of a higher surface area where oxidation of high lignin samples (Figure 7b) resulted in a fragile structure that appears to have been more affected by the oxidation (µm-mm scale) in both the longitudinal direction (Figure 7, upper) and the cross section (Figure 7, lower).

This supports the observation of a more easily fibrillated high lignin wood after oxidation. This was also indicated when handling the wet delignified and oxidized wood samples where high lignin wood was more prone to breaking into smaller pieces. It should further be noted that the porosity induced from removal of lignin from the secondary cell wall was not directly visible during this analysis. Apart from the surface area estimations and large (µm-mm) scale porosity effects that have been shown in this study, direct imaging of nanoscale porosity following TEMPO-oxidation is likely beyond the resolution limit of this analysis.

### 3.4. Outlook and Limitations

In this work, linear regressions between lignin content and presented characteristics have been shown. In many cases a R^2^-value of about 0.7 was apparent, which generally indicates good correlation to initial lignin content. Interesting data points to look at from the context of correlation are the three center points with three samples with approximately 24% initial lignin content. Apparent from the graphs shown throughout is that these tend to fluctuate and interchange in order of best performer. As such, it becomes difficult to correlate strictly between absolute value of lignin content of ease of fibrillation, more than looking at the extreme values of highest and lowest lignin content. The difference between the extremes is more certain and thus the subject of conclusion from this work. The reason behind uncertainty in absolute lignin content and fibrillation likely originate from the biological nature of wood where lignin content can be considered an observable characteristic that originates from the genetic background of the trees that are different across poplars used in this study. Another important emphasis is the choice of treatment that has been used. In this study, direct TEMPO-oxidation was used since it gives the opportunity to minimize the number of experimental steps (and errors) needed to go from wood to final CNFs. With the oxidative nature of the treatment the results are preferably generalized to similar treatments that both removes the lignin and modifies cellulose of the wood.

## 4. Conclusions

Native lignin content in poplar trees (17–30 wt %) was found to impact nanofibrillation and network characteristics following direct TEMPO-oxidation in neutral conditions. Process yields after the treatment ranged between 36 and 44 wt % of initial wood comprising of 45 to 68% nanofibrils with a corresponding viscosity increase between 9 and 27 mPa·s. These fibrillation metrics were higher for high lignin wood compared to low lignin wood whereas surface carboxylation was lower (0.44 mmol·g^−^^1^ and 0.68 mmol·g^−^^1^). The height of the nanofibrils comprising the suspensions ranged between 1.2 and 2.0 nm as measured using AFM.

The dry networks made from the suspensions showed an increased stiffness (3–5 GPa), decreased elongation-at-break (8.2–4.2%) and a similar tensile strength (≈120 MPa) when made from high lignin wood. A higher fraction of char (0.16 to 0.38) was also produced after pyrolysis despite a similar, negligible amount of residual lignin (<1%).

The surface area of TEMPO-oxidized high lignin (30.0 wt %) wood was comparable in the hornified dry state (4.4 and 6.9 m^2^·g^−1^, BET) and higher in wet state (114 vs. 74 m^2^·g^−1^) compared to the low lignin (17.4 wt %). 

Through the direct TEMPO oxidation of wood with a high lignin content, the delignification and carboxylation selectivity of the lignin leads to high porosity which is shown by the increased specific surface area and strongly affected wood ultrastructure. At very high lignin content, this approach effectively makes the wood cell wall more susceptible to disintegration, which in turn can be utilized to achieve a higher degree of fibrillation.

These findings highlight wood with high lignin content as being attractive for CNF isolation and challenges the general viewpoint that lignin is an impediment for processing of wood into CNFs. This study opens for further academic and commercial interest, where i) direct oxidation of wood is novel in the sense of quantifying wood suitability for nanofibrillation and ii) large scale processing can be investigated to look at the potential benefits of a similar lignin variation in a more upscaled commercial environment. 

## Figures and Tables

**Table 1:** Composition of the wood feedstock used in this study shown in wt %.

Sample ID	Coding	Carbohydrates	Extractives	Total Lignin	Cellulose ^1^
1	Low	79.4	0.4	17.4	45 (4)
2		77.2	0.4	19.7	42 (3)
3	Medium	72.4	0.7	24.4	41 (2)
4		72.3	0.5	24.5	40 (3)
5		72.5	0.6	24.6	41 (2)
6	High	66.9	0.7	30.0	39 (2)

^1^ as determined using Test Methods T 203 C TAPPI-standard [15] with three technical replicates on the basis of dried chlorite holocellulose.

**Table 2:** Physical properties of the dry networks made from CNF suspensions. Data represents mean ± SD of six different network samples (*n* = 3 replicates per sample).

Sample ID	Moisture (wt %)	Density (g·cm^−3^)	Thickness (µm)	Porosity (%)	α-cellulose (wt %)	Lignin (wt %)
1	9.1 (1.1)	1.20 (0.10)	71 (10)	20.0 (6.7)	74	<1
2	7.9 (0.8)	1.23 (0.07)	63 (4)	18.0 (4.7)	71	<1
3	7.8 (1.3)	1.19 (0.09)	88 (7)	20.7 (6.0)	76	<1
4	6.8 (1.3)	1.26 (0.10)	50 (5)	16.0 (6.7)	70	<1
5	8.0 (1.4)	1.17 (0.11)	65 (6)	22.0 (7.3)	69	<1
6	7.5 (0.5)	1.20 (0.09)	78 (10)	20.0 (6.0)	76	<1

**Table 3:** Surface area, pore volume and pore size of oxidized wood samples (*n* = 1) as estimated in the freeze-dried state (BET) and in a never-dried state (Congo red).

Sample ID	Coding	BET Surface Area (m^2^g^−1^)	Pore Volume (cm^3^·kg^−1^)	Pore Size (nm)	Wet Surface Area (m^2^g^−1^)
1	Low	6.9	7.5	4.4	76
6	High	4.4	5.7	5.0	114

## Data Availability

Data presented in this study are available on request from the corresponding author.

## Supplementary Materials

The following are available online at https://www.mdpi.com/article/10.3390/nano11051179/s1, supplementary.docx: Figure S1: PCA of trees available in the library. Table S1: Composition of the trees as obtained from pyrolysis. Table S2: Library ID of trees studied in the manuscript.
