# Supplementary material for: The Effect of High Lignin Content on Oxidative Nanofibrillation of Wood Cell Wall
Source: Nanomaterials (Basel). 2021 Apr 29;11(5):1179. doi: 10.3390/nano11051179 (PMC8146676; doi:10.3390/nano11051179)
Supplement: Supplementary file 1 [file nanomaterials-11-01179-s001.zip › nanomaterials-1174633-supplementary.pdf]

Supplementary material

The Effect of High Lignin Content on Oxidative Nanofibrillation of Wood Cell Wall

Simon Jonasson [1], Anne Bünder [2], Linn Berglund [1], Magnus Hertzberg [3] Totte Niittylä [2] and Kristiina Oksman [1,4,*]

1 Division of Materials Science Department of Engineering Sciences and Mathematics, Luleå University of Technology, SE 97187 Luleå, Sweden; simon.jonasson@ltu.se (S.J.); linn.berglund@ltu.se (L.B.); kristiina.oksman@ltu.se (K.O.)

2 Umeå Plant Science Centre, Department of Forest Genetics and Plant Physiology, Swedish University of Agricultural Sciences, SE 90187 Umeå, Sweden; anne.bunder@slu.se (A.B.); totte.niittyla@slu.se (T.N.)

3 SweTree Technologies AB, SE 90403 Umeå, Sweden; magnus.hertzberg@swetree.com

4 Department of Mechanical & Industrial Engineering, University of Toronto, Toronto, ON M5S 3G8, Canada; kristiina.oksman@ltu.se

* Correspondence: kristiina.oksman@ltu.se, telephone +46 920 497731

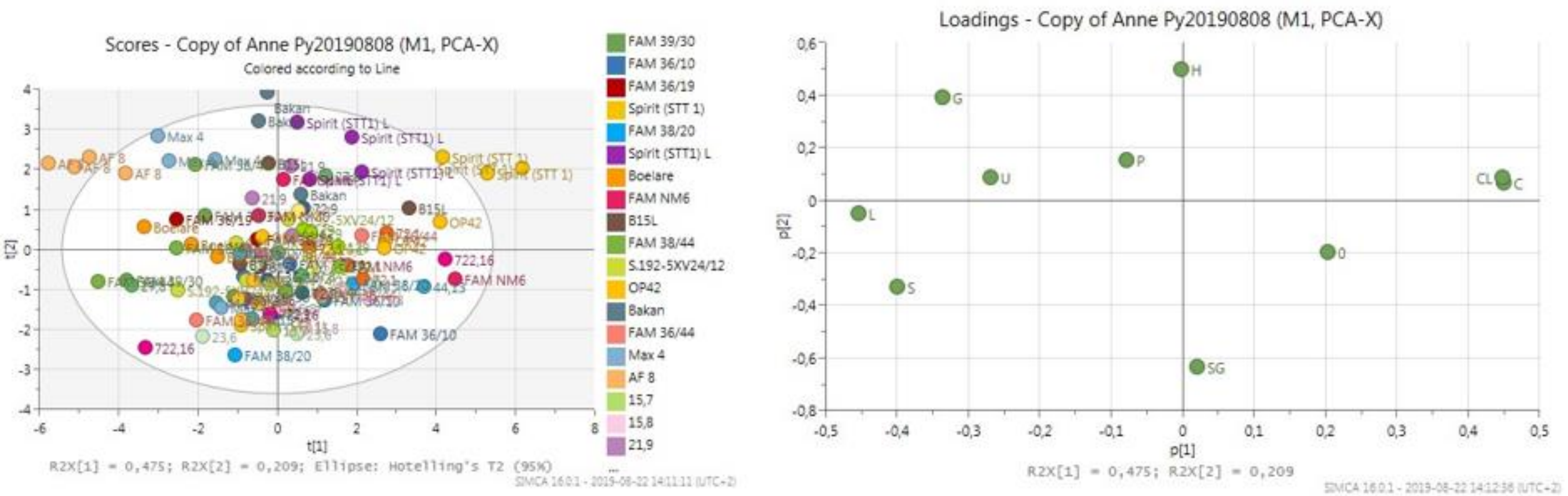

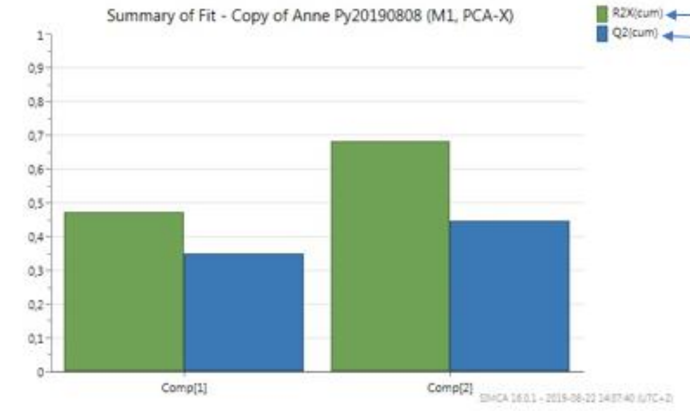

C	Carbohydrate related	Expressed as % of total (C+G+S+H+P+U+0)
G	Guaiacyl	Expressed as % of total (C+G+S+H+P+U+0)
S	Syringyl	Expressed as % of total (C+G+S+H+P+U+0)
H	p-hydroxyphenol	Expressed as % of total (C+G+S+H+P+U+0)
P	Generic Benzen derivatives (without OH group on aromatic ring)	Expressed as % of total (C+G+S+H+P+U+0)
U	known spectra, unknown identification	Expressed as % of total (C+G+S+H+P+U+0)
0	unknown spectra (cannot be lignin or carbohydrates)	Expressed as % of total (C+G+S+H+P+U+0)
SG	S/G ratio	
CL	C/L ratio	
L	total lignin, G+S+H+P	

Figure S1. PCA of trees available in the library.

Table S1. Composition of the trees as obtained from pyrolysis.

Families with clones		Carbohydrate related	G-lignin	S-lignin	H-lignin	Total Lignin
**Spirit (STT 1)**	43.2	79.39413058	5.13556123	11.3382633	0.479532715	17.35833032
Spirit (STT 1)	35.10	77.90872278	5.67030876	12.1201371	0.478696423	18.73974673
Spirit (STT 1)	39.8	77.09619465	6.20669186	12.7300603	0.479924724	19.90349769
Spirit (STT 1)	17.11	70.00975745	5.99208837	19.893098	0.260621655	26.71023274
Spirit (STT1) L	Lugnet 1	73.86934094	6.96649858	14.2871844	0.782496169	22.65626909
Spirit (STT1) L	Lugnet 2	74.14434161	6.65169331	14.6109207	0.583140054	22.47788245
Spirit (STT1) L	Lugnet 3	73.07077676	7.06540946	15.5894921	0.469330069	23.76180161
Spirit (STT1) L	Lugnet 4	72.38398534	7.40979841	15.3957273	0.889546923	24.41310677
Bakan	34.6	72.7408638	6.83737508	16.0513809	0.482324673	23.96943221
Bakan	43.9	72.44773372	7.16687213	15.6758708	1.159890524	24.62900438
Bakan	24.7	70.2446694	6.11923182	19.4662896	0.341748524	26.52696704
Bakan	7.7	71.05757304	7.05783914	16.8110824	1.183449518	25.83318707
15.8	30.9	73.71833999	5.50287681	16.5716549	0.247367014	22.7833253
15.8	23.7	72.25923324	5.37282356	18.3342522	0.23716256	24.45261294
15.8	38.6	71.76451634	5.70574466	18.5744868	0.245609113	25.10671757
15.8	17.12	73.25168045	5.54513301	17.0314725	0.272375223	23.48355789
AF 8	41.11	66.94417967	9.827678	19.1778049	0.343543556	30.02292106
AF 8	12.9	65.16073262	10.6184291	19.9303762	0.50563808	31.69005912
AF 8	9.4	64.89249936	10.0785239	20.8465248	0.559053223	32.20274843
AF 8	35.8	64.40048316	10.8973174	20.6978491	0.389656211	32.63229518
OP42	35.12	74.72830901	6.14658049	15.0107642	0.245130657	21.82841363
OP42	44.10	76.68249215	5.81155258	13.6540381	0.227076194	20.13494716
OP42	25.3	74.99913141	5.93310421	15.1370032	0.177027698	21.77146552
OP42	12.8	71.39227056	7.06777032	17.594932	0.253974536	25.51543051
**Families with individual Genotypes**						
FAM NM6	31.11	77.16978376	4.71932233	14.323105	0.203728997	19.69059518
FAM NM6	46.9	72.512597	7.26972281	16.1644725	0.504119573	24.40969567
FAM 36/10	49.2	74.38439352	4.83883573	16.7587762	0.184183465	22.26252018

Table S2. Library ID of trees studied in the manuscript.

Manuscript ID	Library ID
1	Spirit (STT 1) 43.2
2	FAM NM6 31.11
3	Spirit (STT1)L Lugnet 4
4	15.8 23.7
5	Bakan 43.9
6	AF 8 41.11
